# Gastric acid challenge of lithium disilicate–reinforced glass–ceramics and zirconia-reinforced lithium silicate glass–ceramic after polishing and glazing—impact on surface properties

**DOI:** 10.1007/s00784-023-05301-x

**Published:** 2023-10-11

**Authors:** Jenni Hjerppe, Khalil Shahramian, Emil Rosqvist, Lippo V. J. Lassila, Jouko Peltonen, Timo O. Närhi

**Affiliations:** 1 Clinic of Reconstructive Dentistry, Center of Dental Medicine, University of Zürich, Plattenstrasse 11, 8032 Zurich, Switzerland; 2 Department of Prosthetic Dentistry and Stomatognathic Physiology, University of Turku, Lemminkäisenkatu 2, 20520 Turku, Finland; 3 Physical Chemistry, Laboratory of Molecular Science and Engineering, Åbo Akademi University, Henriksgatan 2, 20500 Turku, Finland; 4 Laboratory Manager, Turku Clinical Biomaterials Centre (TCBC), University of Turku, Itäinen Pitkäkatu 4B, 20520 Turku, Finland; 5 City of Turku, Welfare Division, Lemminkäisenkatu 2, 20520 Turku, Finland; 6 Wellbeing Services County of Southwest Finland, PO BOX 52, 20521 Turku, Finland

**Keywords:** Glass–ceramic, Simulated gastric acid, Surface topography, Surface roughness, Surface microhardness, Corrosion resistance, Stomic force microscopy

## Abstract

**Objectives:**

To investigate the impact of simulated gastric acid on the surface properties of lithium disilicate–reinforced glass–ceramics and zirconia-reinforced lithium silicate glass–ceramic after certain polishing and glazing procedures.

**Materials and methods:**

Four different types of square-shaped specimens (10 × 10 × 2 mm^3^, *n* = 13) were manufactured: lithium disilicate–reinforced glass–ceramic milled and polished (LDS-P); milled, polished, and glazed (LDS-PG); milled, glazed, and no polishing (LDS-G); and milled and polished zirconia-reinforced lithium silicate glass–ceramic (ZR-LS). Specimens were immersed in hydrochloride acid (HCl 0.06 M, pH 1.2) to simulate gastric acid irritation and stored in the acid for 96 h in 37 °C. Specimen weight, surface gloss, Vickers surface microhardness and surface roughness (*R*_a_, *R*_q_, with optical profilometer), and surface roughness on nanometer level (*S*_q_, *S*_al_, *S*_q_/*S*_al_, *S*_dr_, *S*_ds_ with atomic force microscope) were measured before and after the acid immersion.

**Results:**

ZR-LS specimens lost significantly more weight after acid immersion (*p* = 0.001), also surface microhardness of ZR-LS was significantly reduced (*p* = 0.001). LDS-G and LDS-PG showed significantly lower surface roughness (*S*_a_, *S*_q_) values compared to LDS-P before (*p* ≤ 0.99) and after (*p* ≤ 0.99) acid immersion and ZR-LS after acid immersion (*p* ≤ 0.99).

**Conclusions:**

Gastric acid challenge affects the surface properties of lithium disilicate–reinforced glass–ceramic and zirconia-reinforced lithium silicate glass–ceramic. Glazing layer provides lower surface roughness, and the glazed surface tends to smoothen after the gastric acid challenge.

**Clinical relevance:**

Surface finish of lithium disilicate–reinforced glass–ceramic and zirconia-reinforced lithium silicate glass–ceramic has a clear impact on material’s surface properties. Gastric acidic challenge changes surface properties but glazing seems to function as a protective barrier. Nevertheless, also glazing tends to smoothen after heavy gastric acid challenge. Glazing can be highly recommended to all glass–ceramic restorations but especially in patients with gastroesophageal reflux disease (GERD) and eating disorders like bulimia nervosa.

**Supplementary Information:**

The online version contains supplementary material available at 10.1007/s00784-023-05301-x.

## Introduction

Erosive tooth wear is a multifactorial problem caused by the combination of chemical dissolution of tooth structures by acids and mechanical wear of the surfaces thereafter [1]. Acidity of the diet, i.e., soft drink consumption, is an external risk indicator for erosive tooth wear, whereas gastric acid is considered as an internal risk indicator [2–5].

Typical causes of intraoral gastric acid challenges are eating disorders like bulimia nervosa, gastroesophageal reflux disease (GERD), and nausea during pregnancy. GERD is a common medical problem all around the world. Its prevalence varies between 2.5 and 28.8%, being highest in Europe and the USA [6, 7]. Whereas a recent review article was reporting the lifetime prevalence of bulimia nervosa to be 0.63% (95% CI, 0.33–1.02) [8].

Besides erosive tooth wear, high acidity levels intraorally can affect the surface properties of restorative materials [9, 10]. In bulk-fill resin composite materials, gastric acid challenge has shown to increase surface roughness, decrease surface microhardness, and affect the material color [11–13]. On the other hand, with computer-aided design and computer-aided manufacturing (CAD/CAM) resin composite materials, the surface roughness seems to decrease after gastric acid challenge, whereas surface microhardness values do not change [14]. An in vitro study comparing different generations of zirconia materials and milled lithium disilicate–reinforced glass–ceramic showed that lithium disilicate exhibited significantly more weight loss after gastric acid immersion compared to zirconia and the surface roughness decreased in some of the study materials after acid immersion [15]. A decrease of surface roughness has also been seen in an in vitro study comparing the surface properties of monolithic and polished reinforced glass–ceramic and hybrid ceramic materials after gastric acid immersion [16].

Development of monolithic CAD/CAM materials has enabled the efficient chair-side workflow in dental practices. Especially lithium disilicate–reinforced glass–ceramic materials are widely used chair-side due to their great mechanical and esthetic properties as well as straightforward fabrication methods [17–19]. These monolithic materials can be used as polished or as glazed. However, the importance of finalizing procedures of lithium disilicate–reinforced glass–ceramic materials is not fully known when considering the possible surface changes during the gastric acid challenge. Therefore, the aim of this study was to investigate the impact of simulated gastric acid challenge on the surface properties of lithium disilicate–reinforced glass–ceramics and zirconia-reinforced lithium silicate glass–ceramic after polishing and glazing procedures. The null hypothesis was that there is no difference in the surface characteristics and parameters between the groups or within each group before and after the acid immersion.

## Materials and methods

### Specimen preparation

Fifty-two square-shaped specimens (10 × 10 × 2 mm^3^) were manufactured and divided into the following groups (*n* = 13):

- LDS-G: milled lithium disilicate–reinforced glass–ceramic (e.max CAD, Ivoclar Vivadent, Schaan, Lichtenstein), glazed, and no polishing
- LDS-P: milled lithium disilicate–reinforced glass–ceramic (e.max CAD, Ivoclar Vivadent, Schaan, Lichtenstein), polished, and no glazing
- LDS-PG: milled lithium disilicate–reinforced glass–ceramic (e.max CAD, Ivoclar Vivadent, Schaan, Lichtenstein), polished, and glazed
- ZR-LS: milled zirconia-reinforced lithium silicate glass–ceramic (Vita Suprinity, Vita Zahnfabrik GmbH, Bad Säckingen, Germany), polished, and no glazing

Detailed information about the materials is found in Table 1. Specimens in groups LDS-G, LDS-P, LDS-PG, and ZR-LS were milled with a high-speed saw (Struers Secotom-50, Copenhagen, Denmark).

**Table 1:** Detailed information about the studied materials

Materials commercial name	Content*	Manufacturer	Lot no
Vita Suprinity	ZrO_2_ 8–12 wt% SiO_2_ 56–64 wt% Li_2_O 15–21 wt% La_2_O_3_ 0.1 wt% Pigments < 10 wt% Various components > 10 wt%	Vita Zahnfabrik GmbH, Bad Säckingen, Germany	36852
e.max CAD	SiO_2_ 57–80 wt% Li_2_O 11–19 wt% K_2_O 0–13 wt% P_2_O_5_ 0–11 wt% ZrO_2_ 0–8 wt% ZnO 0–8 wt% Al_2_O_3_ 0–5 wt% MgO 0–5 wt% Coloring oxides 0–8 wt%	Ivoclar Vivadent, Schaan, Lichtenstein	T05845
e.max CAD Crystall/Glaze Spray	Oxides Propanol Isobutane	Ivoclar Vivadent, Schaan, Lichtenstein	V26577
Variolink Esthetic DC	Monomer mixture 30–38 wt% Fillers 60–68 wt% Initiators and stabilizers 1–2 wt% Pigments < 1 wt%	Ivoclar Vivadent, Schaan, Lichtenstein	T34190

*Material content information is provided by the manufacturers

After cutting, the specimens (except group LDS-G) were polished on both sides, with silicon carbide paper of grit P1200, 2000, 2400, and 4000 (Struers, Copenhagen, Denmark). Thereafter, a low-speed handpiece (Ultimate XL, NSK Europe GmbH, Eschborn, Germany) was used to polish both sides of the specimens with specific rubber points meant for ceramic polishing (Optrafine, Ivoclar Vivadent, Schaan, Lichtenstein). Polishing was finalized with diamond paste (Brinell L, Renfert GmbH, Hilzingen, Germany) and polishing brushes (HP, Universal, NTI-Kahla GmbH, Kahla, Germany). Specimens were then steam cleaned and crystallized according to manufacturers’ instructions in a specific furnace (Programat 300, Ivoclar Vivadent, Schaan, Lichtestein). After the crystallization procedure, the specimens in the groups LDS-PG and LDS-G were glazed with a glazing spray (e.max CAD Crystall/Glaze Spray, Ivoclar Vivadent). Two layers of glazing material were sprayed onto the surface of the specimens, and the glaze was fired in the porcelain firing oven according to manufacturers’ instructions. The study groups and processing details are summarized in Table 2.

**Table 2:** The ceramic study groups and processing methods

Group	LDS-G	LDS-P	LDS-PG	ZR-LS
Material	Milled lithium disilicate glass–ceramic, glazed	Milled lithium disilicate glass–ceramic, polished	Milled lithium disilicate glass–ceramic, polished, and glazed	Zirconia-reinforced lithium silicate glass- ceramic
Polishing before crystallization/glazing	-	Polished wet with P1200, 2000, 2400, and 4000 grit silicon carbide paper (Struers, Copenhagen, Denmark); polished with rubber tips and diamond paste (Brinell L, Renfert GmbH, Hilzingen, Germany)	Polished wet with P1200, 2000, 2400, and 4000 grit silicon carbide paper (Struers, Copenhagen, Denmark); polished with rubber tips and diamond paste (Brinell L, Renfert GmbH, Hilzingen, Germany)	Polished wet with P1200, 2000, 2400, and 4000 grit silicon carbide paper (Struers, Copenhagen, Denmark); polished with rubber tips (Optrafine, Ivoclar Vivadent, Schaan, Lichtenstein)
Polishing after milling/crystallization	**-**	**-**	**-**	Polished with rubber tips (Optrafine, Ivoclar Vivadent, Schaan, Lichtenstein) and diamond paste (Brinell L, Renfert GmbH, Hilzingen, Germany)

### Scanning electron microscopy

**Fig. 1:**
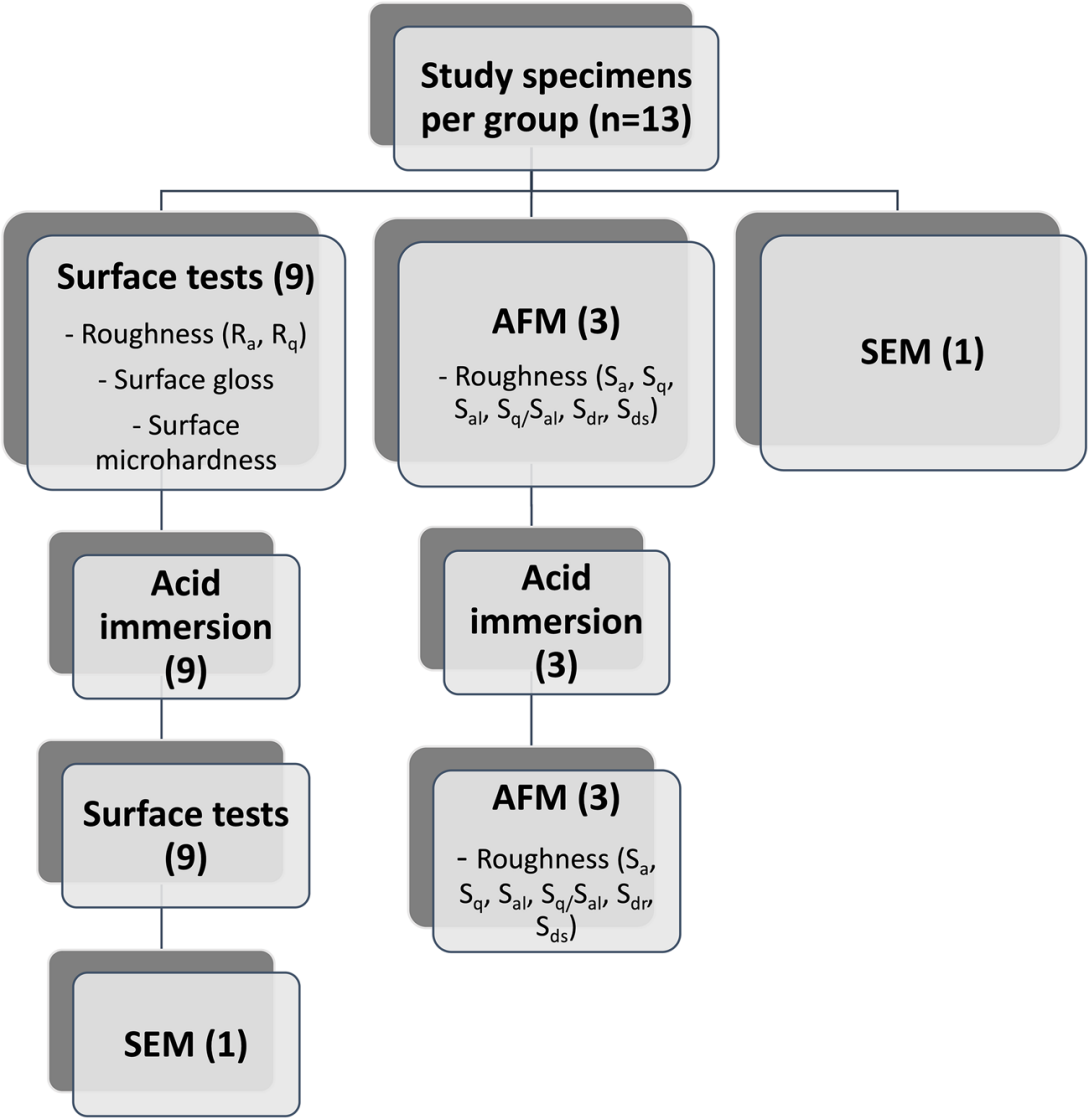
Flowchart of the study protocol for each group (groups ZR-LS, LDS-P, LDS-G, LDS-PG). Abbreviations: LDS-P, polished lithium disilicate–reinforced glass–ceramic; LDS-G, glazed lithium disilicate–reinforced glass–ceramic; LDS-PG, polished and glazed lithium disilicate–reinforced glass–ceramic; ZR-LS, zirconia-reinforced lithium silicate glass–ceramic; AFM, atomic force microscope; SEM, scanning electron microscope; *R*_a_, arithmetic average roughness; *R*_q_, root mean square roughness; *S*_a_, arithmetic average roughness; *S*_q_, root mean square roughness; *S*_al_, autocorrelation length; *S*_q_/*S*_al_, normalized roughness; *S*_dr_, developed surface area ratio; *S*_ds_, density of summits; *S*_ks_, skewness; *S*_ku_, kurtosis

Before acid immersion, one specimen/group was coated with carbon (Bal-Tec SCD 050, Sputter coater), and a field-emission scanning electronic microscope (FE-SEM) (Apreo S, Thermo Scientific, Netherlands) was used for evaluating the morphological changes on the surface (square-shaped specimens). Magnifications of × 300, × 1000, and × 10,000 were used. After the acid immersion and surface tests (roughness, hardness, and gloss measurements), one further specimen/group was sputter-coated with carbon and analyzed with SEM. The study protocol of specimens is illustrated in detail in Fig. 1.

### Acid immersion and weight measurement of the specimens

The specimens (*n* = 9) were immersed in hydrochloric acid (HCl 0.06 M, pH 1.2) to simulate gastric acid irritation in a clinical situation, according to a previously published protocol [15]. The specimens were stored in the acid for 96 h in an incubator (Termaks, Bergen, Norway) at a temperature of 37 °C, and the pH was monitored every 24 h. Furthermore, each specimen was weighted (Mettler Toledo, Columbus, OH, USA) before and after immersion in acid. After the acid immersion, the specimens were rinsed with distilled water, and weights were measured consequently over days to reach a stabilized value.

### Surface gloss measurements

The surface gloss (GU) of 9 specimens/group was measured before and after acid immersion at an incidence angle of 60°, using a calibrated infrared Zehntner Glossmeter (GmbH Testing Instruments, Darmstadt, Germany) with a square measurement area of 6 mm × 40 mm area. Two measurements were recorded per specimen.

### Surface microhardness measurements

A universal Vickers device (Struers Duramin, Struers, Ballerup, Denmark), with a load of 2.94 N being applied for 10 s, was used to measure the surface microhardness of 9 specimens/group before and after acid immersion. Four measurements per specimen were completed. The length of the diagonal of each indentation created by the indenter was measured directly using a graduated eye-lens. The Vickers hardness number (VHN) was obtained using the following equation:

$$VHN=\frac{0.1891\times F}{{d}^{2}}$$

where *VHN* is Vickers hardness number, *F* is the load (N), and *d* is the length of the diagonal (mm).

### Surface roughness measurements

A three-dimensional (3D) non-contact optical profilometer (Contour GT-K, Bruker, Billerica, MA, USA) using Vision64 software (Bruker, Billerica, MA, USA) was used to observe and capture images of the surfaces of 9 specimens/group before and after acid treatment on a micrometer level at × 10, × 20, and × 40 magnification. The surface roughness parameters *R*_a_ (arithmetic average roughness) and *R*_q_ (root mean square (RMS) roughness) (µm) based on line profiles were recorded. Furthermore, the surface topography of the specimens was imaged on a nanometer level before and after the acid immersion using a Nanoscope V MultiMode 8 atomic force microscope (AFM) (Bruker, Billerica, MA, USA) with the peak force mode. Images captured were of 5 µm by 5 µm size with a 512 by 512 pixels resolution. Silicon cantilevers with a nominal tip radius of curvature of 6 nm and a typical spring constant of approx. A 5.1 N/m (NSG01, NT-MDT, Russia) was used for imaging.

Eight different roughness parameters were calculated from the captured AFM images, using the Nanoscope Analysis software (v1.50, Bruker, Billerica, MA, USA). Images were flattened and plane fitted prior to the calculation of the roughness parameters. Prior to these include (arithmetic) average height variation from the mean level of the surface, often called “average roughness” (*S*_a_); RMS average of the height variations from the mean level of the surface, often called “RMS roughness” (*S*_q_); autocorrelation length (*S*_al_); the mean surface slope or the normalized roughness (*S*_q_/*S*_al_), which compares the vertical and lateral distribution of heights [20–23] developed surface area ratio (*S*_dr_); and density of summits (*S*_ds_). The average roughness (*S*_a_) gives the arithmetic average of the height difference at each measurement point to the average height level. The RMS roughness (*S*_q_) is the square root of the sum of all height points’ difference in z-direction from the mean height, i.e., the standard deviation of surface heights. *S*_q_ gives a measure of the vertical height variations of a topographical image. *S*_a_ is commonly used in many standards, but *S*_q_ is considered more statistically robust [24]. The correlation length from the autocorrelation function (*S*_al_) is a measure of the lateral spacing between surface features, the definition being the length over which the correlation function reduces to 20%, i.e., 1/e, of its initial height at origin [24, 25]. The normalized roughness, *S*_q_/*S*_al_, is thus a measure of the ratio between height and lateral variations. The surface area ratio, *S*_dr_, gives the percentual increase in the interfacial area compared to the projected areas, i.e., how much the surface area is increased as a result of the topography compared to a completely flat surface. The summit density, *S*_ds_, is the number of summits per unit area. Summits are defined as peaks (points higher than all adjacent neighboring points) separated by at least 1% of the minimum lateral dimension of the measurement area and are above a threshold that is 5% of height span above the mean plane. Additionally, *S*_pd_, an ISO 25178 replacement of *S*_ds_, data is given in the SI, Figure S1. The *S*_pd_ parameter was calculated after a line-by-line correction using MountainsSPIP version 9.3.10393 (DigitalSurf, Besancon, France).

### Statistical analysis

Statistical analysis was performed with a software SPSS v. 27.0 (IBM SPSS v. 27.0; Chicago, IL, USA). Levene’s test was applied to assess the equality of variance, and the data was analyzed using one-way ANOVA followed by the Tukey HSD post hoc test (surface roughness, weight loss, and surface hardness as dependent variables and specimen type as an independent variable). Student’s *t*-test was used to measure the difference in mean weight loss within each group, before and after acid immersion. A *p*-value of < 0.05 was considered statistically significant.

## Results

### Specimen’s weight loss

The mean weight loss of the study specimens during the acid attack is shown in Table 3. Overall, the weight loss of ceramic materials was minimal, ranging between 0.0005 and 0.0087 wt%, but significant differences between the groups could be seen. ZR-LS material lost significantly more weight compared to pure lithium disilicate specimens (LDS-P, LDS-PG, and LDS-G) after acid immersion (*p* = 0.001). Within groups, a statistically significant weight loss was observed in ZR-LS material (*p* = 0.003).

**Table 3:** Average weight loss (%) of the different study groups after acid immersion

Group	Weight loss % (SD)
LDS-G	0.0005% (0.00003)^a^
LDS-P	0.0020% (0.00004)^a^
LDS-PG	0.0012% (0.00003)^a^
ZR-LS	0.0087% (0.00009)^b^*

Different letters describe significant difference between the groups before and after acid immersion

*Weight loss in group ZR-LS was significantly more compared to other groups (*p* < 0.05)

*LDS-P* polished lithium disilicate–reinforced glass–ceramic, *LDS-G* glazed lithium disilicate–reinforced glass–ceramic, *LDS-PG* polished and glazed lithium disilicate–reinforced glass–ceramic, *ZR-LS* zirconia-reinforced lithium silicate glass–ceramic

### Scanning electron microscopy

**Fig. 2:**
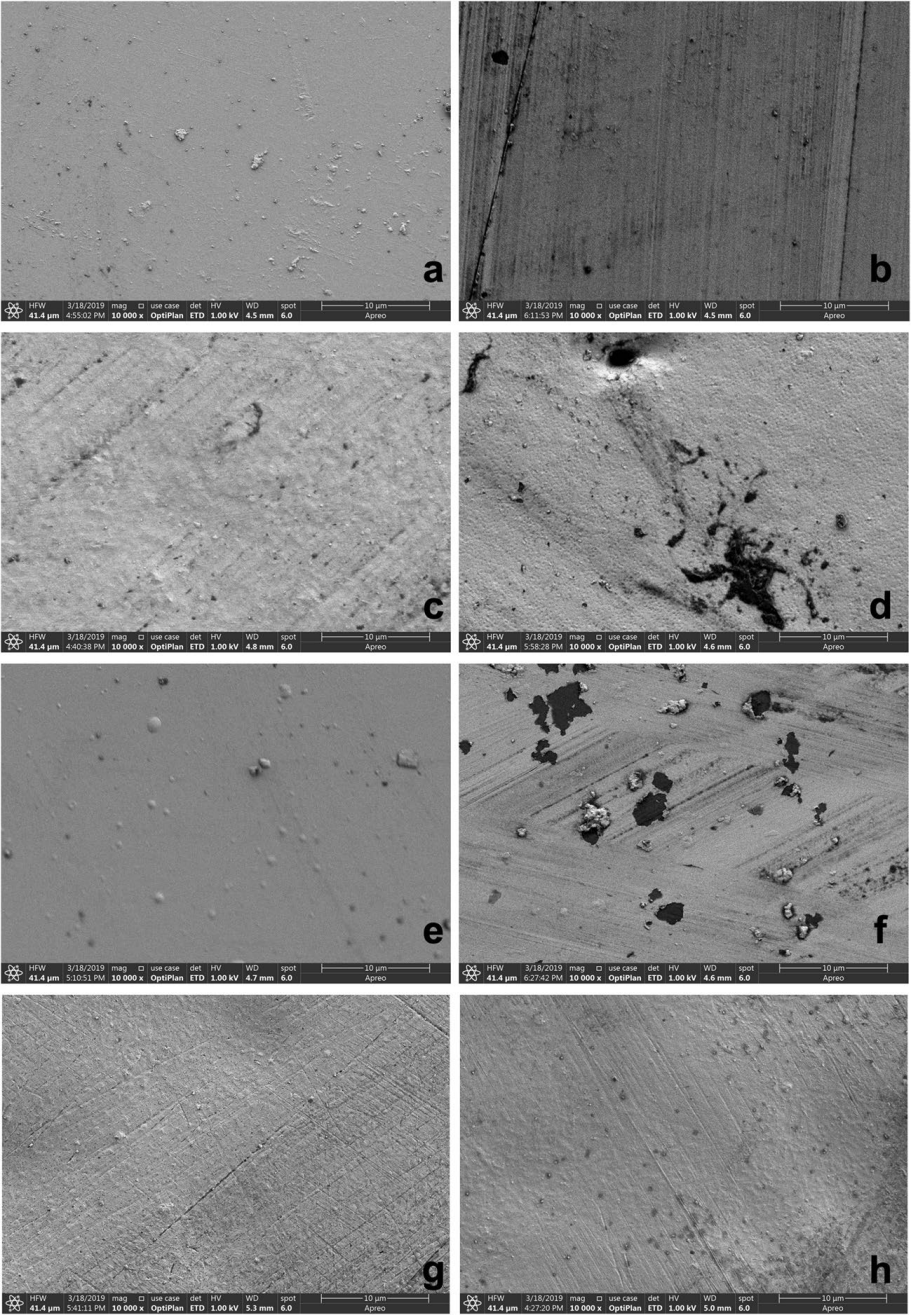
Representative SEM-images of the study groups before and after the acid immersion with magnification of × 10,000. **a** LDS-G before acid immersion, **b** LDS-G after acid immersion, **c** LDS-P before acid immersion, **d** LDS-P after acid immersion, **e** LDS-PG before acid immersion, **f** LDS-PG after acid immersion, **g** ZR-LS before acid immersion, and **h** ZR-LS after acid immersion. Abbreviations: SEM, scanning electron microscope; LDS-P, polished lithium disilicate–reinforced glass–ceramic; LDS-G, glazed lithium disilicate–reinforced glass–ceramic; LDS-PG, polished and glazed lithium disilicate–reinforced glass–ceramic; ZR-LS, zirconia-reinforced lithium silicate glass–ceramic

The surface characteristics of the specimens before and after acid immersion were evaluated with SEM images. Visual inspection showed porosity on LDS-P and ZR-LS specimens’ surfaces after acid immersion (Fig. 2a–h). For LDS-PG and LDS-G, no porosity was observed.

### Surface gloss

The results from surface gloss measurements are presented in Table 4. There was no statistically significant difference in surface gloss before and after acid challenge within any of the study groups (*p* = 0.520). However, the surface gloss of the different groups differed significantly from one another (*p* = 0.001), glazed specimens (LDS-G and LDS-PG) showing higher gloss values compared to the non-glazed groups (LDS-P and ZR-LS).

**Table 4:** Mean surface gloss (GU) values (SD) of the study groups before and after acid immersion

Group	GU before acid (SD)	Statistical differences*	GU after acid (SD)	Statistical differences*
LDS-G	67.2 (18.98)	A	74.2 (11.02)	a
LDS-P	56.1 (15.62)	B	50.5 (14.64)	b
LDS-PG	74.5 (8.39)	A	74.8 (9.98)	a
ZR-LS	58.5 (14.06)	B	51.0 (13.92)	b

*Different letters describe significant difference between the groups, *p* = 0.001

*GU* surface gloss, *LDS-P* polished lithium disilicate–reinforced glass–ceramic, *LDS-G* glazed lithium disilicate–reinforced glass–ceramic, *LDS-PG* polished and glazed lithium disilicate–reinforced glass–ceramic, *ZR-LS* zirconia-reinforced lithium silicate glass–ceramic

### Surface microhardness

**Fig. 3:**
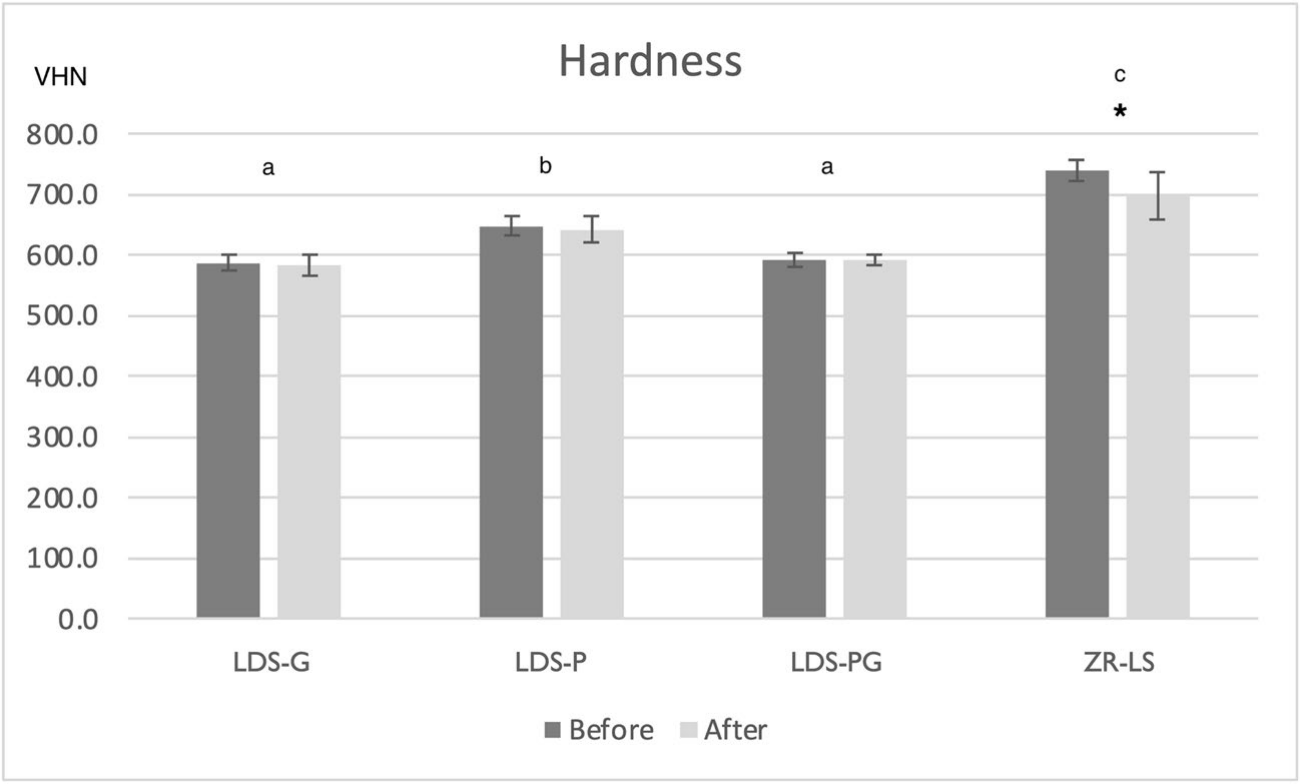
Mean surface microhardness (VHN) and standard deviations of the study specimens before and after acid immersion. *Represents significant difference between the values within the group (*p* = 0.001). Different letters describe significant difference between the groups before and after acid immersion (*p* = 0.001). Abbreviations: LDS-P, polished lithium disilicate–reinforced glass–ceramic; LDS-G, glazed lithium disilicate–reinforced glass–ceramic; LDS-PG, polished and glazed lithium disilicate–reinforced glass–ceramic; ZR-LS, zirconia-reinforced lithium silicate glass–ceramic; VHN, Vickers hardness number

Surface microhardness of ZR-LS was significantly reduced after acid immersion (*p* = 0.001). No difference was observed within the other test groups before and after acid challenge. Furthermore, between the groups, the surface microhardness of LDS-P and ZR-LS groups was significantly higher than those of LDS-G and LDS-PG (*p* = 0.001) (Fig. 3).

### Surface roughness

**Fig. 4:**
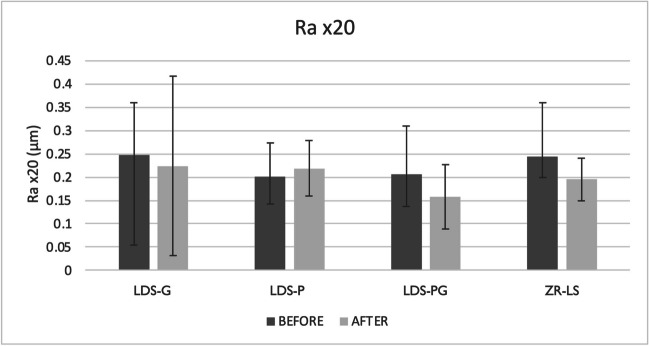
Mean surface roughness *R*_a_ (μm) values and standard deviations determined with optical profilometer with magnification of × 10 before and after acid immersion. Abbreviations: LDS-P, polished lithium disilicate–reinforced glass–ceramic; LDS-G, glazed lithium disilicate–reinforced glass–ceramic; LDS-PG, polished and glazed lithium disilicate–reinforced glass–ceramic; ZR-LS, zirconia-reinforced lithium silicate glass–ceramic; *R*_a_, arithmetic average roughness

Observations with optical profilometer on the roughness of the surfaces are presented in Table 5 and Fig. 4. At magnification level × 10, there were significant differences between the *R*_a_ values of the groups, with CAD-G specimens showing higher surface roughness compared to LDS-P and ZR-LS specimens (*p* = 0.001). In addition, within each group, the *R*_a_ values after acid treatment were significantly different from the ones before acid treatment (*p* = 0.048). This was also true for the *R*_q_ values at magnification level × 10 (*p* = 0.026), showing a trend of decreasing surface roughness after acid challenge. At × 20 magnification, no differences between the groups or within a group were seen (*p* > 0.05). At × 40 magnification, *R*_a_ values of the groups were significantly different from each other, with glazed specimens (LDS-G and LDS-PG) showing significantly lower surface roughness compared to LDS-P and ZR-LS groups (*p* = 0.001). *R*_q_ values of the groups were also significantly different from one another (*p* = 0.001). No difference within a group before and after acid treatment was observed at this magnification level (*p* = 0.583 for *R*_a_ and *p* = 0.494 for *R*_q_ values).

**Table 5:** Mean surface roughness *R*_a_ and *R*_q_ values in μm (with SD) of the study groups before and after acid immersion with magnifications of × 10, × 20, × 40

Surface roughness					
*R*_a_ × 10	Before acid	After acid	*R*_q_ × 10	Before acid	After acid
LDS-G	0.50 (0.2)	0.38 (0.1)	LDS-G	0.60 (0.3)	0.48 (0.2)
LDS-P	0.25 (0.1)	0.24 (0.1)	LDS-P	0.38 (0.3)	0.33 (0.1)
LDS-PG	0.41 (0.3)	0.31 (0.1)	LDS-PG	0.50 (0.3)	0.39 (0.2)
ZR-LS	0.30 (0.1)	0.23 (0.1)	ZR-LS	0.59 (0.5)	0.32 (0.1)
*R*_a_ × 20	Before acid	After acid	*R*_q_ × 20	Before acid	After acid
LDS-G	0.25 (0.1)	0.22 (0.2)	LDS-G	0.30 (0.1)	0.28 (0.2)
LDS-P	0.20 (0.1)	0.22 (0.1)	LDS-P	0.27 (0.1)	0.28 (0.1)
LDS-PG	0.21 (0.1)	0.16 (0.1)	LDS-PG	0.26 (0.1)	0.20 (0.1)
ZR-LS	0.25 (0.1)	0.20 (0.05)	ZR-LS	0.32 (0.2)	0.26 (0.1)
*R*_a_ × 40	Before acid	After acid	*R*_q_ × 10	Before acid	After acid
LDS-G	0.09 (0.1)	0.08 (0.1)	LDS-G	0.11 (0.1)	0.10 (0.1)
LDS-P	0.18 (0.1)	0.18 (0.1)	LDS-P	0.23 (0.1)	0.23 (0.1)
LDS-PG	0.08 (0.05)	0.06 (0.03)	LDS-PG	0.10 (0.1)	0.07 (0.04)
ZR-LS	0.16 (0.1)	0.17 (0.04)	ZR-LS	0.21 (0.2)	0.21 (0.1)

*LDS-P* polished lithium disilicate–reinforced glass–ceramic, *LDS-G* glazed lithium disilicate–reinforced glass–ceramic, *LDS-PG* polished and glazed lithium disilicate–reinforced glass–ceramic, *ZR-LS* zirconia-reinforced lithium silicate glass–ceramic, *R*_*a*_ arithmetic average roughness, *R*_*q*_ root mean square roughness

AFM measurements of the ceramic specimens’ nano-topography revealed some differences between the groups and within a group before and after acid immersion. Analyzed roughness parameters are summarized in Table 6.. Height roughness parameter (*S*_a_ and *S*_q_) values were varying in the range of 0.43–18.25 nm, respectively, 1.32–22.68 nm, depending on the group. The effective surface area (*S*_dr_) indicated that the surfaces were relatively smooth in all groups, being 0.91–1.49% before and 0.22–2.54% after the acid treatment. The LDS-G and LDS-PG surfaces had both before and after acid treatment a peak dominated nano-topography, the *S*_sk_ being on average 5.39 and 2.74 before and 19.23 and 8.94 after acid treatment. The other specimens’ topographies, LDS-P and ZR-LS, being clearly neither peak nor valley dominated (*S*_sk_ 0.18–0.54).

**Table 6:** Mean values of different surface roughness parameters and their standard deviations before and after acid immersion determined from 5 µm × 5 µm AFM images

Surface roughness (5 µm × 5 µm AFM images)					
*S*_a_ (nm)	Before acid	After acid	*S*_q_ (nm)	Before acid	After acid
LDS-G	3.82 (1.07)	0.43 (0.19)	LDS-G	7.31 (2.77)	1.32 (0.81)
LDS-P	18.25 (1.80)	17.20 (3.51)	LDS-P	22.68 (2.05)	21.56 (4.27)
LDS-PG	3.25 (2.38)	2.84 (3.89)	LDS-PG	5.69 (5.00)	4.11 (4.60)
ZR-LS	6.00 (0.67)	8.21 (0.55)	ZR-LS	7.48 (0.84)	10.20 (0.68)
*S*_al_ (µm)	Before acid	After acid	*S*_q_/*S*_al_ (nm/µm)	Before acid	After acid
LDS-G	0.28 (0.12)	0.12 (0.05)	LDS-G	0.03 (-)	0.01 (-)
LDS-P	0.52 (0.07)	0.53 (0.10)	LDS-P	0.04 (-)	0.04 (-)
LDS-PG	0.27 (0.11)	0.29 (0.31)	LDS-PG	0.02 (-)	0.01 (-)
ZR-LS	0.31 (0.02)	0.30 (0.03)	ZR-LS	0.02 (-)	0.03 (-)
*S*_dr_ (%)	Before acid	After acid	*S*_ds_ (1/µm^2^)	Before acid	After acid
LDS-G	1.49 (0.62)	0.22 (0.17)	LDS-G	16.70 (5.72)	5.53 (4.02)
LDS-P	1.12 (0.43)	2.54 (3.60)	LDS-P	18.58 (6.00)	25.18 (16.80)
LDS-PG	1.31 (1.10)	1.03 (1.90)	LDS-PG	43.37 (32.99)	30.76 (22.94)
ZR-LS	0.91 (0.10)	1.25 (0.11)	ZR-LS	43.70 (3.55)	44.85 (2.07)
*S*_sk_ ( −)	Before acid	After acid	*S*_ku_ ( −)	Before acid	After acid
LDS-G	5.39 (0.38)	19.23 (8.09)	LDS-G	53.35 (8.6)	601 (40)
LDS-P	0.23 (0.34)	0.47 (0.32)	LDS-P	2.95 (0.5)	3.25 (0.7)
LDS-PG	2.74 (2.11)	8.94 (5.86)	LDS-PG	30.5 (28.5)	149 (144)
ZR-LS	0.18 (0.07)	0.54 (0.14)	ZR-LS	3.01 (0.14)	3.07 (0.18)

*LDS-P*, polished lithium disilicate–reinforced glass–ceramic, *LDS-G* glazed lithium disilicate–reinforced glass–ceramic, *LDS-PG* polished and glazed lithium disilicate–reinforced glass–ceramic, *ZR-LS* zirconia-reinforced lithium silicate glass–ceramic, *S*_*a*_ arithmetic average roughness, *S*_*q*_ root mean square roughness, *S*_*al*_ autocorrelation length, *S*_*q*_*/S*_*al*_ normalized roughness, *S*_*dr*_ developed surface area ratio, *S*_*ds*_ density of summits, *S*_*ks*_ skewness, *S*_*ku*_ kurtosis

LDS-G showed an overall smoothening after acid treatment. This was seen as a decrease in *S*_a_ and *S*_q_ (*p* = 0.0016, resp., *p* = 0.0017), as well as in *S*_al_, *S*_dr_, and *S*_ds_ (*p* = 0.042, *p* = 0.011, resp., *p* = 0.034). These show reduced height variations, density of local peaks (fine structure), and effective surface area. Both before and after acid treatment, the surfaces were peak dominated (*S*_sk_ > 0, Table 6.), but the smoothening was seen as a narrower distribution (leptokurtic, *p* = 0.016, Table 6.). The LDS-P specimens did not show a significant change (cutoff *p* = 0.05) in any parameter on this length scale due to the acid treatment. The LDS-PG specimens did not appear to change in any other parameter but the *S*_sk_, which showed a more peak driven topography after the acid treatment (*S*_sk_ increasing from 2.74 to 8.94, *p* = 0.038), indicating some nano-topographical change. The ZR-LS showed an opposite trend with an increasing surface roughness after acid immersion, in terms of height variations (*S*_a_ and *S*_q_) as well as the *S*_dr_ (*p* = 0.0012, *p* = 0.0016, resp., *p* = 0.0029). A change was also observed in the height distribution, indicating an increased peak dominated topography. This was shown by the *S*_sk_, which increased from 0.18 to 0.54 (*p* = 0.0047).

**Fig. 5:**
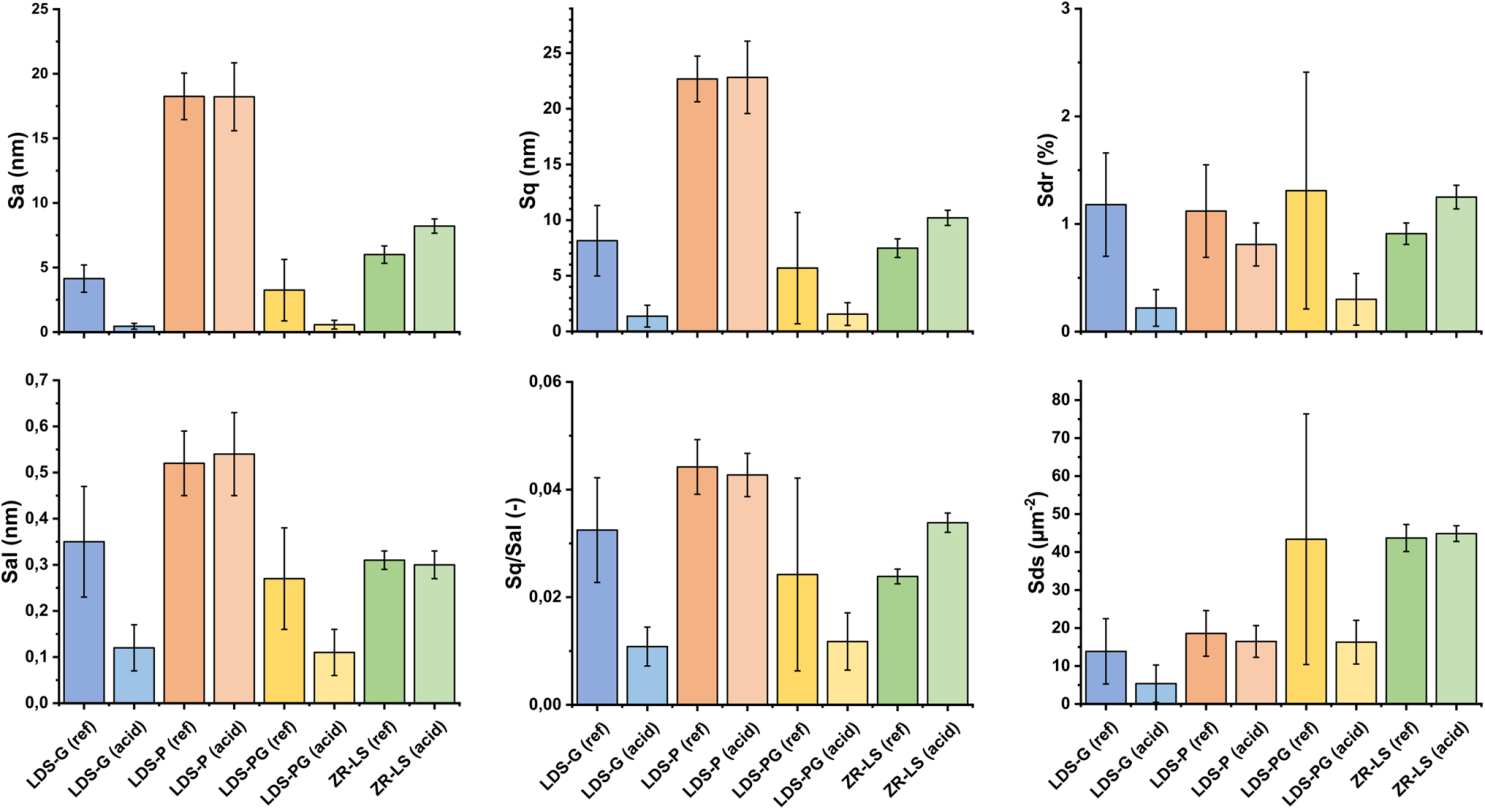
Mean surface roughness values of parameters *S*_a_, *S*_q_, *S*_al_, *S*_q_/*S*_al_, *S*_dr_, and *S*_ds_ (nm) and standard deviations of the specimens, before (ref) and after (acid) the acid immersion, determined with AFM. Abbreviations: LDS-P, polished lithium disilicate–reinforced glass–ceramic; LDS-G, glazed lithium disilicate–reinforced glass–ceramic; LDS-PG, polished and glazed lithium disilicate–reinforced glass–ceramic; ZR-LS, zirconia-reinforced lithium silicate glass–ceramic; AFM, atomic force microscope; *S*_a_, arithmetic average roughness; *S*_q_, root mean square roughness; *S*_al_, autocorrelation length; *S*_q_/*S*_al_, normalized roughness; *S*_dr_, developed surface area ratio; *S*_ds_, density of summits

**Fig. 6:**
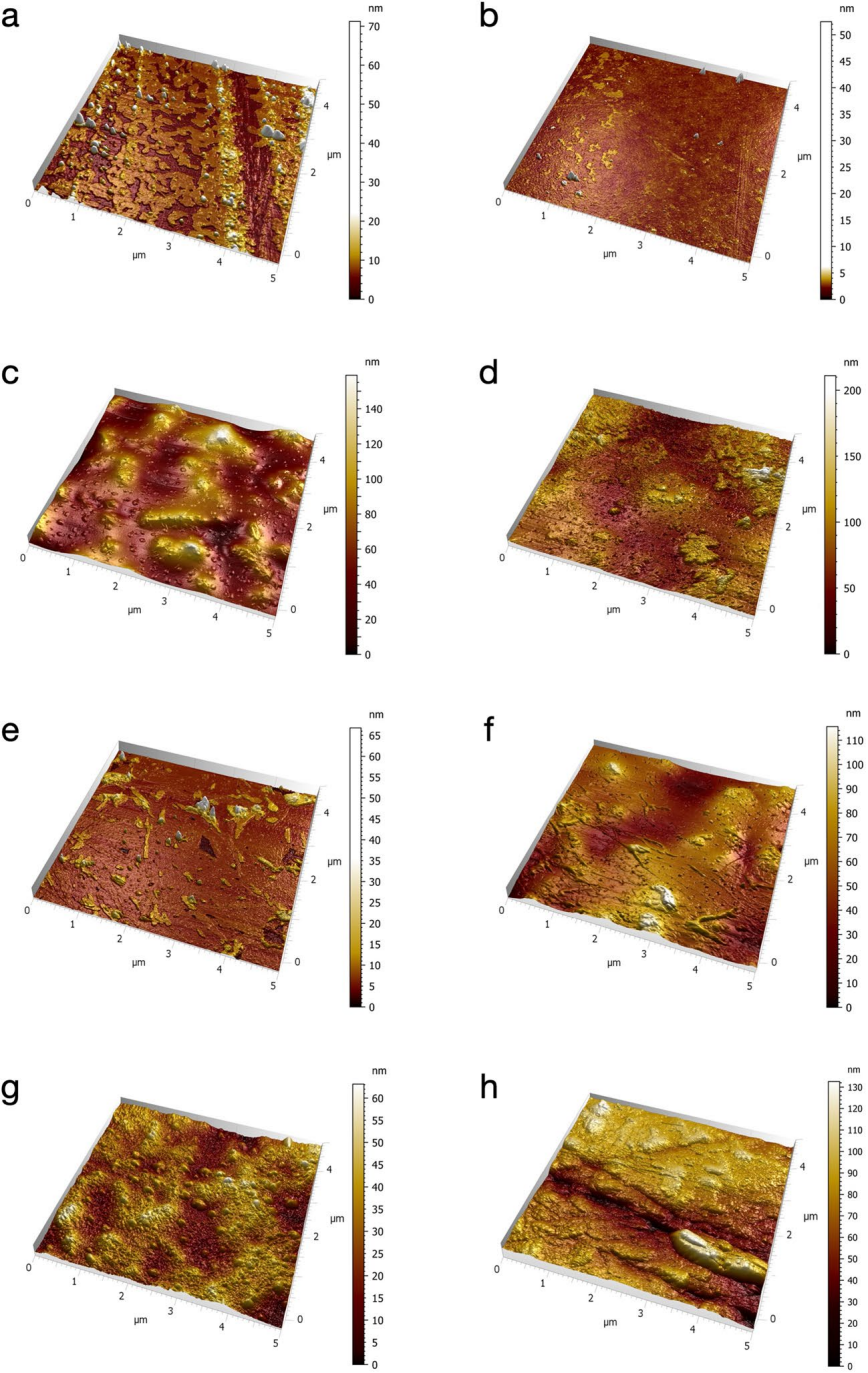
Representative AFM-images (with image *Z*-axis ranges) before and after acid immersion of ceramic study materials: **a** LDS-G before acid (*Z* = 72.55 nm), **b** LDS-G after acid (*Z* = 52.57 nm), **c** LDS-P before acid (*Z* = 164.3 nm), **d** LDS-P after acid (*Z* = 150.7 nm), **e** LDS-PG before acid (*Z* = 66.34 nm), **f** LDS-PG after acid (*Z* = 122.4 nm), **g** ZR-LS before acid (*Z* = 61.76 nm), **h** ZR-LS after acid (*Z* = 117.9 nm). Abbreviations: LDS-P, polished lithium disilicate–reinforced glass–ceramic; LDS-G, glazed lithium disilicate–reinforced glass–ceramic; LDS-PG, polished and glazed lithium disilicate–reinforced glass–ceramic; ZR-LS, zirconia-reinforced lithium silicate glass–ceramic; AFM, atomic force microscope

When comparing height roughness (*S*_a_ and *S*_q_) values between different groups (see Fig. 5 and Figure S1, Table S1 and S2), glazed specimens LDS-G and LDS-PG had significantly lower surface roughness compared to LDS-P before (LDS-G *p*_Sa_ < 0.01, *p*_Sq_ < 0.01; LDS-PG *p*_Sa_ < 0.01, *p*_Sq_ < 0.01) and after (LDS-G *p*_Sa_ < 0.01, *p*_Sq_ < 0.01; LDS-PG *p*_Sa_ < 0.01, *p*_Sq_ < 0.01) acid immersion and to ZR-LS after acid immersion (*p* < 0.01). ZR-LS showed significantly lower *S*_a_ and *S*_q_ values compared to LDS-P before (*p*_Sa_ < 0.01, *p*_Sq_ < 0.01) and after (*p*_Sa_ < 0.01, *p*_Sq_ < 0.01) acid immersion. Overall, there was a trend of decreasing surface roughness after acid immersion (Fig. 5, Figure FS1). For visual inspection, representative AFM images of the ceramic specimens before and after acid immersion are shown in Fig. 6a–h. The relative differences of the specimens in different surface roughness parameters before and after the acid treatment are shown in Fig. 6. In short, LDS-G showed the largest relative change, while LDS-P and LDS-PG changed least. These surfaces all showed some smoothening due to the acid treatment. The ZR-LS surface was the only sample that showed an increase in roughness.

## Discussion

This study aimed to investigate the impact of simulated gastric acid challenge on the surface properties of lithium disilicate–reinforced glass–ceramics and zirconia-reinforced lithium silicate glass–ceramic after polishing and glazing procedures. Each group exhibited weight loss after acid immersion. ZR-LS material lost significantly more weight compared to lithium disilicate specimens. Additionally, the surface gloss, surface microhardness, and surface roughness values on micro and nano level were significantly different among the groups. Differences were also seen when comparing the roughness and microhardness parameters of each group before and after the acid immersion. Therefore, the null hypothesis was rejected.

It is known that the acidic challenge episodes in GERD occur several times per day, and the duration of the episodes can last some hours [26]. The number of acid reflux episodes has shown to correlate to time of which the acid reflux is present in esophagus and the number of dental erosion signs on teeth [27]. The acid challenge seems to have a long-term effect on tooth structures. In order to investigate the long-term effect of the gastric acid on lithium disilicate–reinforced glass–ceramic and zirconia-reinforced lithium silicate glass–ceramic materials, a rather aggressive acid challenge (HCl, pH 1.2, 96 h at 37 °C) was conducted in the present study. This is estimated to correspond to over 10 years of clinical exposure [15]. However, the effect of possible confounding factors, like presence of saliva in a clinical situation, could not be taken into consideration.

Weight loss of the dental restorative materials after acid challenge has been reported in previous studies as well [15, 28, 29]. Exposure time, solution pH, and material type all play a role in the level of surface degradation and weight loss [29]. In the present study, significantly more weight loss after acid challenge was seen in zirconia-reinforced lithium silicate glass–ceramic material (ZR-LS) compared to the lithium disilicate–reinforced glass–ceramic specimens. This might be due to different material microstructures and their grade of dissolution in acidic surroundings [30]. In relation to restorative material wear, a recent in vitro study reported a significantly greater weight loss in human enamel specimens compared to zirconia-reinforced lithium disilicate glass–ceramic [28].

In the present study, the polished specimens (LDS-P, ZR-LS) had higher surface microhardness compared to glazed specimens (LDS-G, LDS-PG). A previous study about microhardness of different ceramic materials has shown that material itself is more decisive in determining the microhardness than acid challenge [16]. They reported no significant differences in surface microhardness after acid challenge. In the present study, after a simulated gastric acid challenge, only in the ZR-LS group was the difference seen within a material, with surface microhardness being significantly lower afterwards. In the present study however, the acid challenge was five times longer, proving again the effect of the exposure time on the results [29]. A recent study comparing the surface parameters of CAD/CAM restorative materials has reported the benefits of polishing the surfaces after erosion cycles [31]. In the study, milled lithium disilicate–reinforced glass–ceramic, hybrid ceramic, and poly methyl methacrylate (PMMA) specimens as well as human enamel specimens were attached to an intraoral appliance and subjected to in situ erosion cycles (rinsing with cola drink). Different surface properties were negatively affected by erosion cycles; however, repolishing of the material or enamel surfaces was found to restore the surface microhardness values.

Roughness parameter analysis provides a quantitative way of analyzing the data by providing measurements of various topographical features and their change. The disadvantage with solely visual inspection of the images is drawing only qualitative and even subjective conclusions. In this study, surface roughness was evaluated with several different parameters. *R*_a_ and *R*_q_ values measured with optical profilometer showed a trend of decreasing surface roughness after acid immersion. Similar results have been also reported in a previous study by Cruz et al. [16]. Due to the differences observed in roughness before and after acid treatment in the present study, additional AFM-analysis was carried out. Arithmetic mean and RMS roughness are two regularly encountered roughness parameters [25, 32]. However, when characterizing a surface, several parameters should be used to obtain an adequate description of its geometry [23]. These parameters add in describing different geometrical aspects of the surface topography—e.g., amplitude, spacing between asperities, and parameters relating to the distribution of heights [32–35]. While the average roughness, *S*_a_, is a common parameter due to its accessibility, the RMS roughness, *S*_q_, is considered statistically more robust [24]. When evaluating and comparing roughness data, one should also be aware of the influence of the tip shape, scale, and image resolution on the obtained parameter values [24, 33]. The present results show that the specimens with glazing layer had lower surface roughness parameters (*S*_a_ and *S*_q_), and the surface was smoothened after the gastric acid challenge (groups LDS-G, LDS-PG). Trend of degreasing surface roughness parameters was also seen in the polished lithium disilicate group (LDS-P), whereas the ZR-LS specimens became rougher.

A rough ceramic surface can be a risk factor for tooth wear of the occluding pair [28, 36]. A glazing or veneering layer is typically added to achieve better esthetic result of the final ceramic restoration. However, it is also shown that veneered lithium disilicate causes more antagonist wear than unveneered material [37]. Based on the results of the present study, glazing layer might be beneficial especially for the patients with a condition involving intra oral gastric acid challenge. A clinically problematic situation might come up after occlusal adjustments of cemented crowns. During the occlusal adjustments, the glazing layer can be worn out, and the exposed ceramic surface can be more prone to changes by acid. In such patients, regular maintenance care and careful polishing of the restorations are of importance. With polishing procedures, some of the surface properties can be restored [31].

This in vitro study evaluated the effect of gastric acid challenge on lithium disilicate–reinforced glass–ceramic and zirconia-reinforced lithium silicate glass–ceramic materials. Another clinical aspect, mechanical wear, was not accomplished and can be considered as a limitation of this study. However, the study is presenting important information about the impact of gastric acid alone. For fabricating the glazed specimens, two layers of special glazing spray were used. The analyses in this study were done on the surface of the specimens, and therefore, the final thickness of the glazing layer is not known. This could be seen as a limitation when interpreting the results. In future studies, thickness of the glazed layer before and after the acid immersion could be analyzed from the cross section of the specimens. Additionally, a hand-made glazing layer might not have been equally thick. This can also explain the small differences in AFM surface roughness parameters between LDS-G and LDS-PG groups. Results of this in vitro study could be validated in prospective clinical study on patients suffering from GERD.

## Conclusions

Gastric acid challenge affects the surface properties of lithium disilicate–reinforced glass–ceramic and zirconia-reinforced lithium silicate glass–ceramic. Glazing layer provides lower surface roughness, and the surface tends to smoothen after the gastric acid challenge.

### Supplementary Information

Below is the link to the electronic supplementary material.

Supplementary file1 (TIF 30925 KB)

Supplementary file2 (DOCX 20 KB)

Supplementary file3 (DOCX 21 KB)

## Data Availability

All data related to this research may be requested from the authors.
