# Supplementary material for: Gastric acid challenge of lithium disilicate–reinforced glass–ceramics and zirconia-reinforced lithium silicate glass–ceramic after polishing and glazing—impact on surface properties
Source: Clin Oral Investig. 2023 Oct 11;27(11):6865–77. doi: 10.1007/s00784-023-05301-x (PMC10630222; doi:10.1007/s00784-023-05301-x)
Supplement: Supplementary file 2 — Supplementary file2 (DOCX 20 KB) [file 784_2023_5301_MOESM2_ESM.docx]

**Supplementary Table S1** — Relative changes in the evaluated roughness parameters after acid immersion.

ZR-LS, zirconia and lithium disilicate reinforced glass-ceramic (Vita Suprinity, Vita Zahnfabrik GmbH), LDS-P, polished lithium disilicate reinforced glass-ceramic (e.max CAD, Ivoclar Vivadent), LDS-G glazed lithium disilicate reinforced glass-ceramic (e.max CAD, e.max Crystall/Glaze Spray, Ivoclar Vivadent), LDS-PG polished and glazed lithium disilicate reinforced glass-ceramic (e.max CAD, e.max Crystall/Glaze Spray, Ivoclar Vivadent), AFM, atomic force microscope, S_a_, arithmetic average roughness, S_q_ root mean squared roughness, S_al_, autocorrelation length, S_q_/S_al_, normalized roughness, S_dr_ developed surface area ratio, S_ds_ density of summits.

| **Param.:** | **LDS-G** | **LDS -P** | **LDS-PG** | **ZR-LS** |
| --- | --- | --- | --- | --- |
| **S_a_** | 0.11 | 0.94 | 0.88 | 1.37 |
| **S_q_** | 0.18 | 0.95 | 0.72 | 1.36 |
| **S_dr_** | 0.14 | 2.27 | 0.79 | 1.38 |
| **S_al_** | 0.43 | 1.02 | 1.07 | 0.97 |
| **S_q_/S_al_** | 0.43 | 0.93 | 0.68 | 1.41 |
| **S_ds_** | 0.33 | 1.35 | 0.71 | 1.03 |
