# Supplementary material for: Gastric acid challenge of lithium disilicate–reinforced glass–ceramics and zirconia-reinforced lithium silicate glass–ceramic after polishing and glazing—impact on surface properties
Source: Clin Oral Investig. 2023 Oct 11;27(11):6865–77. doi: 10.1007/s00784-023-05301-x (PMC10630222; doi:10.1007/s00784-023-05301-x)
Supplement: Supplementary file 3 — Supplementary file3 (DOCX 21 KB) [file 784_2023_5301_MOESM3_ESM.docx]

**Supplementary Table S2** — Relative changes in the evaluated roughness parameters after acid immersion.

|  | **LDS-G** | | | | **LDS-P** | | | |
| --- | --- | --- | --- | --- | --- | --- | --- | --- |
| **Param.** | **Ref** |  | **Acid** |  | **Ref** |  | **Acid** |  |
| **S_a_ (nm)** | 4,14 | 1,06 | 0,45 | 0,23 | 18,25 | 1,8 | 18,22 | 2,63 |
| **S_q_ (nm)** | 8,15 | 3,16 | 1,37 | 0,98 | 22,68 | 2,05 | 22,82 | 3,25 |
| **S_dr_ (%)** | 1,18 | 0,48 | 0,22 | 0,17 | 1,12 | 0,43 | 0,81 | 0,2 |
| **S_al_ (µm)** | 0,35 | 0,12 | 0,12 | 0,05 | 0,52 | 0,07 | 0,54 | 0,09 |
| **S_q_/S_al_ (-)** | 3,25E-02 | 9,73E-03 | 1,08E-02 | 3,61E-03 | 4,42E-02 | 5,07E-03 | 4,27E-02 | 4,02E-03 |
| **S_ds_ (1/µm^2^)** | 13,85 | 8,59 | 5,35 | 4,91 | 18,58 | 6 | 16,47 | 4,18 |
|  |  |  |  |  |  |  |  |  |
|  | **LDS-PG** | | | | **ZR-LS** | | | |
| **Param.** | **Ref** |  | **Acid** |  | **Ref** |  | **Acid** |  |
| **S_a_ (nm)** | 3,25 | 2,38 | 0,57 | 0,34 | 6 | 0,67486 | 8,21 | 0,55 |
| **S_q_ (nm)** | 5,69 | 5 | 1,56 | 1,02 | 7,48 | 0,84 | 10,2 | 0,68 |
| **S_dr_ (%)** | 1,31 | 1,1 | 0,3 | 0,24 | 0,91 | 0,1 | 1,25 | 0,11 |
| **S_al_ (µm)** | 0,27 | 0,11 | 0,11 | 0,05 | 0,31 | 0,02 | 0,3 | 0,03 |
| **S_q_/S_al_ (-)** | 2,42E-02 | 1,79E-02 | 1,18E-02 | 5,32E-03 | 2,39E-02 | 1,36E-03 | 3,38E-02 | 1,80E-03 |
| **S_ds_ (1/µm^2^)** | 43,37 | 32,99 | 16,27 | 5,74 | 43,7 | 3,55 | 44,85 | 2,07 |
